# Spoofing Detection Algorithm Based on Pseudorange Differences

**DOI:** 10.3390/s18103197

**Published:** 2018-09-21

**Authors:** Ke Liu, Wenqi Wu, Zhijia Wu, Lei He, Kanghua Tang

**Affiliations:** 1 College of Artificial Intelligence, National University of Defense Technology, Changsha 410073, China; wenqiwu_lit@hotmail.com(W.W.); wuzhijia94@outlook.com (Z.W.); tt_kanghua@hotmail.com (K.T.); 2 College of Systems Engineering, National University of Defense Technology, Changsha 410073, China; helei_nudt@163.com

**Keywords:** single receiver, meaconing attack, simplistic attack, intermediate attack, anti-spoofing technology, pseudorange difference

## Abstract

Intentional spoofing interference can cause damage to the navigation terminal and threaten the security of a global navigation satellite system (GNSS). For spoofing interference, an anti-spoofing algorithm based on pseudorange differences for a single receiver is proposed, which can be used to detect simplistic and intermediate spoofing attacks, as well as meaconing attacks. Double-difference models using the pseudorange of two adjacent epochs are established followed by the application of Taylor expansion to the position relationship between the satellite and the receiver (or the spoofer). The authenticity of the signal can be verified by comparing the results of the proposed spoofing detection algorithm with the traditional least squares method. The results will differ when spoofing is present. The parameter setting of the proposed algorithm is introduced. The algorithm has the advantage of both simplicity and efficiency and needs only a single receiver and pseudorange data. A NovAtel receiver is adopted for the actual experiments. The Texas spoofing test battery (TEXBAT), as well as two other simulation experiments are used to verify the performance of the algorithm. The simulation results validate the feasibility and effectiveness of the algorithm.

## 1. Introduction

As global satellite navigation systems (GNSSs) play an increasingly important role in society and industry, the security of these systems is a crucial component. Intentional and unintentional spoofing interference affects normal use of navigation and timing terminals. Unlike jamming, the goal of spoofing is to take control of the user receiver. The receiver captures the spoofing signal and uses it for the calculation of an incorrect positioning. In [1], the authors analyzed the vulnerability of the satellite signal and the GNSS to attacks, illustrating how the spoofing signal enters and takes over the receiver. To facilitate a threat analysis, the authors divided the spoofing threat into three categories: simplistic, intermediate and sophisticated spoofing attacks [2]. In addition to these three categories, there is another case: meaconing. Meaconing is the interception and replay of navigation signals on the received frequency, typically with a power higher than the original signal, to confuse the navigation terminal [3].

The main form and the purpose of a spoofer is to generate a similar and false satellite signal and make the receiver capture it. The spoofer is defined as a system containing three elements: the receiving antenna, the spoofing signal generator and the transmitting antenna. The receiving antenna receives the signal and inputs it into the spoofing signal generator. Then, the generator generates the spoofing signal, which has the same form as the satellite signal according to the input and the spoofing interference intention and is finally transmitted by the transmitting antenna. The signal input to the generator and the signal output from the generator are not the same, but are similar. The main form of the meaconer is to delay the transmission of the real signal by the delay module. A meaconer contains the same three elements as the spoofer; however, the meaconer has a signal transponder with a delay module instead of the spoofing signal generator. The whole process can be described as: the receiving antenna receives the signal and inputs it into the signal transponder, and the signal is then transmitted by the transmitting antenna after a certain delay. The signal input into the transponder and the signal output from the transponder are the same. The structure of the signal in this spoofing scenario is not changed. Compared with the spoofer, meaconing has the advantage that it is easier to implement and has a lower cost. The coarse/acquisition code (C/A code) of the satellite signal is open and transparent, and its structure is well known to the public. Therefore, the spoofing signal, which is very similar to the real satellite navigation signal, can mislead the receiver from the correct position. The precision code (P code) is encrypted and cannot be simulated easily. Even so, meaconing still can use it by the time delay module.

For spoofing interference, the spoofer should first destroy the connection between the receiver and the real signal and should then make the receiver capture the spoofing signal, preferably by a higher signal power than the normal one. In [4], the authors point out that the tracking of the real signal can be destroyed as long as the power of the spoofing signal is at least four decibels (dB) higher than the real signal in the condition that the pseudo-code rate difference between the real signal and the spoofing signal is 1/3 Hertz (Hz) and the receiver’s coherent accumulated time is 1 ms. To suppress spoofing interference, the existence of the spoofing signal must first be accurately detected. Previous detection technologies mainly focused on signal distortion detection, by considering the signal power [5,6], the spatial distribution properties [7,8] and by observing the change rates of ranges and the clock offset/drifts [1]. The signal power is influenced by many factors during transmission, and transient power increase does not mean the spoofing signal exists.

Spoofing interference can be simple or complex:

Simplistic spoofing attacks and meaconing can be regarded as “simple spoofing”. This “simple spoofing” is defined as a non-overlapped spoofing scenario. The main characteristic of this kind of spoofing is that the correlation peak of the spoofing pseudo-random noises (PRNs) is not overlapped with that of the authentic ones. This attack is usually generated by a hardware simulator or replayed by a signal transponder. An effective way to spoof a receiver in a non-overlapped scenario is to first jam and make the receiver lose its lock on the real signal and instead capture the spoofing signal [9]. In this scenario, the spoofing signal appears as a noise and only affects the effective carrier noise power ratio (C/No). For example, in the simulation scenario of the “Beidou Open Laboratory test” in Section 3, the signal is switched from the real to the spoofing signal with an absolute power advantage. Another effective way to spoof a receiver in a non-overlapped scenario is that the receiver enters an area that the satellite signal cannot cover, then the signal is generated through the simulator or replayed through the transponder to achieve spoofing. In this case, even if the value of C/No is lower than that of the real signal, the spoofing signal can still be captured by the receiver. In this scenario, the value of C/No is mainly affected by the transmitting power. The simulation scenario of the “university test” in Section 3 is an example of this case.

The simulation scenarios of “the Texas spoofing test battery” used in this paper are more complex and can be regarded as “complex spoofing”. The “complex spoofing” is defined as overlapped spoofing scenarios. In an overlapped spoofing attack, the correlation peak of the spoofing signals and the real signals overlap, and this interaction misshapes the correlation peak. This kind of spoofing attack is generated by a receiver-based spoofing generator where the spoofer knows the current time, the observable satellites and the location and signal parameters of the target receiver. The real signal is separated from the composite signal (which is the overlap between the real signal and the spoofing signal) by a small power advantage to realize the signal switch. This kind of spoofing is harder to detect.

For simple spoofing, some related methods in [1] are valid. However, for complex spoofing, these methods become invalid.

Current research focuses on using the antenna array method [10], the receiver pseudorange or carrier phase difference [11,12], the correlation method [13,14], the inertial aided method [15,16] or the hypothesis testing method [17,18]. The antenna array method needs more than one antenna. Its detection performance is affected by the baseline length between the antennas. When there is only one single receiver or one single antenna, such a difference method cannot be used. The inertial aided method has some disadvantages. First, inertial devices are needed, and this increases the cost of the whole navigation system. Second, inertial navigation involves error accumulation over time. This makes it effective over a short period, but invalid over longer periods. There are also some studies on the hardware of the receiver needed to detect the signal [19,20]. All hardware-based methods require a change of the structure of existing receivers. Signal encryption is also a scheme to avoid spoofing interference, which is analyzed in [21]. However, the implementation of this solution requires a comprehensive and systematic modification from satellite to receiver, which is not feasible in a short time. If users want to use the signal, permission is needed from the operator. Furthermore, as meaconing does not change the signal structure, the scheme of signal encryption cannot effectively suppress meaconing.

In addition, some crossing methods are proposed. In Ref. [22], the authors proposed a method to monitor the spoofing signal based on machine learning and signal processing. Other methods can also detect spoofing signals to a certain extent, but still have limitations. Considering that some of such methods are complementary in spoofing detection, the authors adopted information fusion in combination with multiple spoofing detection strategies to improve the detection performance [23]. The information fusion method is useful, but requires more hardware and software to realize the different detection methods and requires more time to finish the signal processing. The performance of the fusion algorithm determines the effectiveness of detection. In Ref. [24], the authors proposed a network monitoring mechanism based on the time difference of arrival properties between spoofing and authentic signals. This network contains several receivers and one central processing component. From the simulation, we can see that the detection performance is influenced by the distance between the receiver and the central processing component. This structure is better suited for static testing.

If a single receiver can be used to implement detections, the disadvantages mentioned above can be overcome. The motivation of this paper is to use a single receiver or a single antenna to detect meaconing, simplistic and intermediate spoofing attacks by pseudorange differences. The main contributions of this paper can be summarized as follows: (1) we build the signal pseudorange model based on the signal transmission path; (2) a novel spoofing detection algorithm is proposed based on the pseudorange model, which only needs one single receiver and does not require changing the hardware; (3) we validate the proposed algorithm on real experiments, showing its effectiveness and simplicity in real engineering applications. The hardware of the receiver does not need to be changed, and no additional auxiliary equipment is required. This is the advantage compared with other algorithms. The authenticity of the signal can be confirmed by comparing the result of the proposed spoofing detection algorithm with the result of the traditional least squares method. The paper is organized as follows: Section 2 gives the theoretical analysis of a single receiver against the spoofing signal; Section 3 describes three different test datasets, which are used to validate the algorithm performance; Section 4 concludes the paper.

## 2. Spoofing Detection Algorithm

In this section, we introduce the theoretical analysis of the algorithm. We build the single receiver pseudorange double-difference model, then apply Taylor expansion and iterative calculation to the pseudorange double-difference model to obtain the position at the current epoch. By comparing the results of the traditional least squares method to the spoofing detection algorithm proposed in this paper, we can identify the authenticity of the signal. The parameter setting of the proposed algorithm is pointed out in Section 2.2. The algorithm summary and explicit flowchart are given in Section 2.3.

### 2.1. Theoretical Analysis

First, we define the distance between $X^{i}$ and $X_{p}$ as:

(1) $$R\left(X^{i},X_{p}\right)=\sqrt{{\left(x^{i}-x_{p}\right)}^{2}+{\left(y^{i}-y_{p}\right)}^{2}+{\left(z^{i}-z_{p}\right)}^{2}}$$

where $X^{i}=\left(x^{i},y^{i},z^{i}\right)$ represents the satellite position in the Earth-centered Earth-fixed (ECEF) coordinates and $X_{p}=\left(x_{p},y_{p},z_{p}\right)$ represents the vehicle position in the ECEF coordinates.

Suppose the pseudorange of the ith satellite at $t_{k}$ is $\rho_{k}^{i}$ and at $t_{k+1}$ is $\rho_{k+1}^{i}$. We have:

(2) $$\rho_{k}^{i}=R\left(X_{k}^{i},X_{p,k}\right)+\left(\delta t_{r,k}-\delta t_{s,k}^{i}\right)\times c+\left(\delta t_{ion,k}^{i}+\delta t_{trop,k}^{i}\right)\times c+\varepsilon_{k}^{i}$$

(3) $$\rho_{k+1}^{i}=R\left(X_{k+1}^{i},X_{p,k+1}\right)+\left(\delta t_{r,k+1}-\delta t_{s,k+1}^{i}\right)\times c+\left(\delta t_{ion,k+1}^{i}+\delta t_{trop,k+1}^{i}\right)\times c+\varepsilon_{k+1}^{i}$$

where $\delta t_{r,m}$ is the receiver clock offset at $t_{m}$, $\delta t_{s,m}^{i}$ is the ith satellite clock offset at $t_{m}$, $\delta t_{ion,m}^{i}$ is the ith satellite ionosphere delay at $t_{m}$, $\delta t_{trop,m}^{i}$ is the ith satellite troposphere delay at $t_{m}$, *c* is the speed of light and $\varepsilon_{m}^{i}$ is the ith satellite non-model errors, such as the measurement noise at $t_{m}$.

We use the ionosphere delay correction, the troposphere delay correction and the satellite clock offset correction to correct the pseudorange. Then, the pseudorange single difference of Equations (2) and (3) is:

(4) $$\Delta \rho_{k+1,k}^{i}=\rho_{k+1}^{i}-\rho_{k}^{i}=R\left(X_{k+1}^{i},X_{p,k+1}\right)-R\left(X_{k}^{i},X_{p,k}\right)+\left(\delta t_{r,k+1}-\delta t_{r,k}\right)\times c+\left(\varepsilon c_{k+1}^{i}-\varepsilon c_{k}^{i}\right)$$

where $\varepsilon c_{m}^{i}$ represents the ith satellite’s non-model errors, such as the measurement noise and the ionosphere delay, the troposphere delay and the satellite clock offset correction residuals at $t_{m}$.

Similarly, for the jth satellite, we have:

(5) $$\Delta \rho_{k+1,k}^{j}=\rho_{k+1}^{j}-\rho_{k}^{j}=R\left(X_{k+1}^{j},X_{p,k+1}\right)-R\left(X_{k}^{j},X_{p,k}\right)+\left(\delta t_{r,k+1}-\delta t_{r,k}\right)\times c+\left(\varepsilon c_{k+1}^{j}-\varepsilon c_{k}^{j}\right)$$

The pseudorange double difference is calculated between Equations (4) and (5). Equation (6) is the pseudorange double-difference model.

(6) $$\begin{array}{cc} \Delta \rho_{k+1,k}^{ij} & =\Delta \rho_{k+1,k}^{i}-\Delta \rho_{k+1,k}^{j}\\ & =\;\left[\;R\;\left(\;X_{k+1}^{i},X_{p,k+1}\;\right)\;\;-\;R\;\left(\;X_{k}^{i},X_{p,k}\;\right)\;\;\right]\;\;-\;\;\left[\;R\;\left(\;X_{k+1}^{j},X_{p,k+1}\;\right)\;\;-\;R\;\left(\;X_{k}^{j},X_{p,k}\;\right)\;\;\right]\;\;+\;\;\left(\;\varepsilon c_{k+1}^{i}\;-\;\varepsilon c_{k}^{i}\;\right)\;\;-\;\;\left(\;\varepsilon c_{k+1}^{j}\;-\;\varepsilon c_{k}^{j}\;\right)\; \end{array}$$

In Equation (6), the distances $R\left(X_{k}^{i},X_{p,k}\right)$ and $R\left(X_{k}^{j},X_{p,k}\right)$ are known when we detect the authenticity of the signal at $t_{k+1}$, so Taylor expansion is only needed for $R\left(X_{k+1}^{i},X_{p,k+1}\right)$ and $R\left(X_{k+1}^{j},X_{p,k+1}\right)$.

Taylor expansion of $R\left(X_{k+1}^{i},X_{p,k+1}\right)$ is done at $\left(x_{k},y_{k},z_{k}\right)$. We have:

(7) $$R\left(X_{k+1}^{i},X_{p,k+1}\right)\approx R\left(X_{k+1}^{i},X_{k}\right)+u_{x,k+1,k}^{i}\Delta x+u_{y,k+1,k}^{i}\Delta y+u_{z,k+1,k}^{i}\Delta z$$

where,

$$u_{x,k+1,k}^{i}=-\left(x_{k+1}^{i}-x_{k}\right)/R\left(X_{k+1}^{i},X_{k}\right)$$

$$u_{y,k+1,k}^{i}=-\left(y_{k+1}^{i}-y_{k}\right)/R\left(X_{k+1}^{i},X_{k}\right)$$

$$u_{z,k+1,k}^{i}=-\left(z_{k+1}^{i}-z_{k}\right)/R\left(X_{k+1}^{i},X_{k}\right)$$

$$\left\{\begin{array}{c} x_{k+1,SDA}=x_{k}+\Delta x\\ y_{k+1,SDA}=y_{k}+\Delta y\\ z_{k+1,SDA}=z_{k}+\Delta z \end{array}\right.$$

where $u_{k+1,k}^{i}=\left[\begin{array}{ccc} u_{x,k+1,k}^{i} & u_{y,k+1,k}^{i} & u_{z,k+1,k}^{i} \end{array}\right]$ is the line-of-sight unit vector of the ith satellite. The subscripts *k* and k+1 represent the time point $t_{k}$ and $t_{k+1}$, respectively. $\left(x_{k+1,SDA},y_{k+1,SDA},z_{k+1,SDA}\right)$ is the position vector at $t_{k+1}$, and it is the quantity we need to calculate by the algorithm proposed in this paper.

Similarly, Taylor expansion of $R\left(X_{k+1}^{j},X_{p,k+1}\right)$ is done at $\left(x_{k},y_{k},z_{k}\right)$. The two expansion equations are substituted into Equation (6), then we have:

(8) $$\begin{array}{c} R\left(X_{k+1}^{i},X_{k}\right)\;-\;R\left(X_{k+1}^{j},X_{k}\right)\;+\;u_{x,k+1,k}^{i}\left(x_{k+1,SDA}\;-\;x_{k}\right)\;+\;u_{y,k+1,k}^{i}\left(y_{k+1,SDA}\;-\;y_{k}\right)\;+\;u_{z,k+1,k}^{i}\left(z_{k+1,SDA}\;-\;z_{k}\right)\\ -u_{x,k+1,k}^{j}\left(x_{k+1,SDA}-x_{k}\right)-u_{y,k+1,k}^{j}\left(y_{k+1,SDA}-y_{k}\right)-u_{z,k+1,k}^{j}\left(z_{k+1,SDA}-z_{k}\right)\\ =\Delta \rho_{k+1,k}^{i}-\Delta \rho_{k+1,k}^{j}+\left[R\left(X_{k}^{i},X_{p,k}\right)-R\left(X_{k}^{j},X_{p,k}\right)\right]-\left(\varepsilon c_{k+1}^{i}-\varepsilon c_{k}^{i}\right)+\left(\varepsilon c_{k+1}^{j}-\varepsilon c_{k}^{j}\right) \end{array}$$

If there are *n* satellites with the same PRN number at $t_{k}$ and $t_{k+1}$, the pseudorange double difference is calculated between the first satellite and the other $\left(n-1\right)$ satellites, respectively. Therefore, $\left(n-1\right)$ equations are obtained. Taylor expansion is applied to these equations and written in the matrix form.

(9) $$\begin{array}{c} M\left[\begin{array}{c} x_{k+1,SDA}-x_{k}\\ y_{k+1,SDA}-y_{k}\\ z_{k+1,SDA}-z_{k} \end{array}\right]=L \end{array}$$

where,

$$\begin{array}{cc} M & =M_{1}-M_{2}\\ & =\left[\begin{array}{ccc} u_{x,k+1,k}^{1} & u_{y,k+1,k}^{1} & u_{z,k+1,k}^{1}\\ u_{x,k+1,k}^{1} & u_{y,k+1,k}^{1} & u_{z,k+1,k}^{1}\\ \vdots & \vdots & \vdots \\ u_{x,k+1,k}^{1} & u_{y,k+1,k}^{1} & u_{z,k+1,k}^{1} \end{array}\right]-\left[\begin{array}{ccc} u_{x,k+1,k}^{2} & u_{y,k+1,k}^{2} & u_{z,k+1,k}^{2}\\ u_{x,k+1,k}^{3} & u_{y,k+1,k}^{3} & u_{z,k+1,k}^{3}\\ \vdots & \vdots & \vdots \\ u_{x,k+1,k}^{n} & u_{y,k+1,k}^{n} & u_{z,k+1,k}^{n} \end{array}\right] \end{array}$$

$$\begin{array}{cc} L & =L_{1}-L_{2}\\ & =\;\left[\;\begin{array}{c} \Delta \rho_{k+1,k}^{1}\;+\;R\left(X_{k}^{1},X_{p,k}\right)\;-\;R\left(X_{k+1}^{1},X_{k}\right)\;-\;\left(\varepsilon c_{k+1}^{1}\;-\;\varepsilon c_{k}^{1}\right)\\ \Delta \rho_{k+1,k}^{1}\;+\;R\left(X_{k}^{1},X_{p,k}\right)\;-\;R\left(X_{k+1}^{1},X_{k}\right)\;-\;\left(\varepsilon c_{k+1}^{1}\;-\;\varepsilon c_{k}^{1}\right)\\ \vdots \\ \Delta \rho_{k+1,k}^{1}\;+\;R\left(X_{k}^{1},X_{p,k}\right)\;-\;R\left(X_{k+1}^{1},X_{k}\right)\;-\;\left(\varepsilon c_{k+1}^{1}\;-\;\varepsilon c_{k}^{1}\right) \end{array}\;\right]\;\\ \; & -\;\;\left[\;\begin{array}{c} \Delta \rho_{k+1,k}^{2}\;+\;R\left(X_{k}^{2},X_{p,k}\right)\;-\;R\left(X_{k+1}^{2},X_{k}\right)\;-\;\left(\varepsilon c_{k+1}^{2}\;-\;\varepsilon c_{k}^{2}\right)\\ \Delta \rho_{k+1,k}^{3}\;+\;R\left(X_{k}^{3},X_{p,k}\right)\;-\;R\left(X_{k+1}^{3},X_{k}\right)\;-\;\left(\varepsilon c_{k+1}^{3}\;-\;\varepsilon c_{k}^{3}\right)\\ \vdots \\ \Delta \rho_{k+1,k}^{n}\;+\;R\left(X_{k}^{n},X_{p,k}\right)\;-\;R\left(X_{k+1}^{n},X_{k}\right)\;-\;\left(\varepsilon c_{k+1}^{n}\;-\;\varepsilon c_{k}^{n}\right) \end{array}\;\right]\; \end{array}$$

The size of M is $\left(n-1\right)\times 3$, and the size of L is $\left(n-1\right)\times 1$. To compare the differences between the position vector of the spoofing detection algorithm proposed in this paper and the position vector of the traditional least squares method in the analytic expression, we add a row to the matrix M and L, respectively. Equation (9) can then be rewritten as:

(10) $$\left\{\left[\begin{array}{c} 0_{1\times 3}^{M}\\ M_{1} \end{array}\right]-\left[\begin{array}{c} 0_{1\times 3}^{M}\\ M_{2} \end{array}\right]\right\}\left[\begin{array}{c} x_{k+1,SDA}-x_{k}\\ y_{k+1,SDA}-y_{k}\\ z_{k+1,SDA}-z_{k} \end{array}\right]=\left\{\left[\begin{array}{c} 0_{1\times 1}^{L}\\ L_{1} \end{array}\right]-\left[\begin{array}{c} 0_{1\times 1}^{L}\\ L_{2} \end{array}\right]\right\}$$

where,

$$0_{1\times 3}^{M}=\left[\begin{array}{ccc} u_{x,k+1,k}^{1} & u_{y,k+1,k}^{1} & u_{z,k+1,k}^{1} \end{array}\right]$$

$$0_{1\times 1}^{L}=\left[\Delta \rho_{k+1,k}^{1}+R\left(X_{k}^{1},X_{p,k}\right)-R\left(X_{k+1}^{1},X_{k}\right)-\left(\varepsilon c_{k+1}^{1}-\varepsilon c_{k}^{1}\right)\right]$$

As the following relationships exist:

(11) $$\left[\begin{array}{c} 0_{1\times 3}^{M}\\ M_{1} \end{array}\right]\left[\begin{array}{c} x_{k+1,SDA}-x_{k}\\ y_{k+1,SDA}-y_{k}\\ z_{k+1,SDA}-z_{k} \end{array}\right]=\left[\begin{array}{c} \rho_{k+1}^{1}-R\left(X_{k+1}^{1},X_{k}\right)-\delta t_{r,k+1}\times c-\varepsilon c_{k+1}^{1}\\ \rho_{k+1}^{1}-R\left(X_{k+1}^{1},X_{k}\right)-\delta t_{r,k+1}\times c-\varepsilon c_{k+1}^{1}\\ \vdots \\ \rho_{k+1}^{1}-R\left(X_{k+1}^{1},X_{k}\right)-\delta t_{r,k+1}\times c-\varepsilon c_{k+1}^{1} \end{array}\right]$$

(12) $$\left[\rho_{k}^{i}-R\left(X_{k}^{i},X_{p,k}\right)-\varepsilon c_{k}^{i}\right]-\left[\rho_{k}^{j}-R\left(X_{k}^{j},X_{p,k}\right)-\varepsilon c_{k}^{j}\right]=0$$

We calculate the difference between $\left\{\left[\begin{array}{c} 0_{1\times 1}^{L}\\ L_{1} \end{array}\right]-\left[\begin{array}{c} 0_{1\times 1}^{L}\\ L_{2} \end{array}\right]\right\}$ and $\left[\begin{array}{c} 0_{1\times 3}^{M}\\ M_{1} \end{array}\right]\left[\begin{array}{c} x_{k+1,SDA}-x_{k}\\ y_{k+1,SDA}-y_{k}\\ z_{k+1,SDA}-z_{k} \end{array}\right]$. Combining the obtained difference result with Equations (11) and (12), Equation (10) can be rewritten as:

(13) $$\;\left[\;\begin{array}{c} 0_{1\times 3}^{M}\\ M_{2} \end{array}\;\right]\;\;\left[\;\begin{array}{c} x_{k+1,SDA}\\ y_{k+1,SDA}\\ z_{k+1,SDA} \end{array}\;\right]\;\;=\;\;\left[\;\begin{array}{c} 0_{1\times 3}^{M}\\ M_{2} \end{array}\;\right]\;\;\left[\;\begin{array}{c} x_{k}\\ y_{k}\\ z_{k} \end{array}\;\right]\;\;+\;\;\left[\;\begin{array}{c} \rho_{k+1}^{1}-R\left(X_{k+1}^{1},X_{k}\right)\\ \rho_{k+1}^{2}-R\left(X_{k+1}^{2},X_{k}\right)\\ \vdots \\ \rho_{k+1}^{n}-R\left(X_{k+1}^{n},X_{k}\right) \end{array}\;\right]\;\;-\;\;\left[\;\begin{array}{c} \delta t_{r,k+1}\times c\\ \delta t_{r,k+1}\times c\\ \vdots \\ \delta t_{r,k+1}\times c \end{array}\;\right]\;\;-\;\;\left[\;\begin{array}{c} \varepsilon c_{k+1}^{1}\\ \varepsilon c_{k+1}^{2}\\ \vdots \\ \varepsilon c_{k+1}^{n} \end{array}\;\right]\;$$

By calculating Equation (13) iteratively, the position vector $\left(x_{k+1,SDA},y_{k+1,SDA},z_{k+1,SDA}\right)$ at $t_{k+1}$ for the spoofing detection algorithm can be obtained.

For the traditional least squares method [25], only the measurement information of one time epoch is needed. The pseudorange after corrections at $t_{k+1}$ can be written as:

(14) $$\rho_{k+1}^{i}=R\left(X_{k+1}^{i},X_{p,k+1}\right)+\delta t_{r,k+1}\times c+\varepsilon c_{k+1}^{i}$$

Taylor expansion for $R\left(X_{k+1}^{i},X_{p,k+1}\right)$ is done at $\left(x_{k},y_{k},z_{k}\right)$. Then, we have:

(15) $$G_{e}\left[\begin{array}{c} x_{k+1,LS}\\ y_{k+1,LS}\\ z_{k+1,LS}\\ \delta t_{r,k+1}\times c \end{array}\right]=G_{e}\left[\begin{array}{c} x_{k}\\ y_{k}\\ z_{k}\\ 0 \end{array}\right]+\left[\begin{array}{c} \rho_{k+1}^{1}-R\left(X_{k+1}^{1},X_{k}\right)\\ \rho_{k+1}^{2}-R\left(X_{k+1}^{2},X_{k}\right)\\ \vdots \\ \rho_{k+1}^{n}-R\left(X_{k+1}^{n},X_{k}\right) \end{array}\right]-\left[\begin{array}{c} \varepsilon c_{k+1}^{1}\\ \varepsilon c_{k+1}^{2}\\ \vdots \\ \varepsilon c_{k+1}^{n} \end{array}\right]$$

where,

$$G_{e}=\left[\begin{array}{cc} 0_{1\times 3}^{M} & 1_{1\times 1}\\ M_{2} & 1_{\left(n-1\right)\times 1} \end{array}\right]$$

By calculating Equation (15) iteratively, the position vector $\left(x_{k+1,LS},y_{k+1,LS},z_{k+1,LS}\right)$ at $t_{k+1}$ for the traditional least squares method can be obtained.

**Figure 1:**
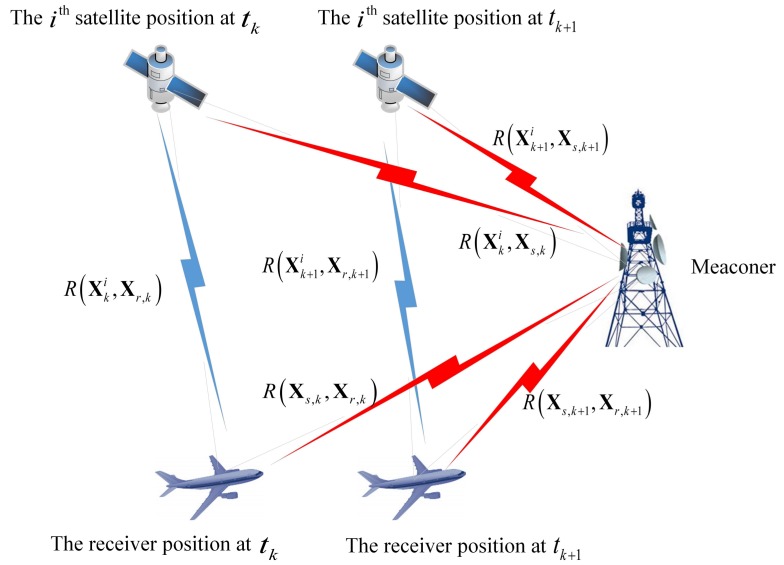
The signal transmission path.

According to the signal transmission path presented in Figure 1, we can build the pseudorange model. Then, we have:

- When the signal is real, it transmits directly from the satellite to the receiver. We have $R\left(X_{k+1}^{i},X_{p,k+1}\right)=R\left(X_{k+1}^{i},X_{r,k+1}\right)$.
- When the signal is spoofing, according to the principle of the spoofing attack, we have: (1) for the meaconer, it includes the physical distance between the satellite and the meaconer $R\left(X_{k+1}^{i},X_{s,k+1}\right)$, and the physical distance between the meaconer and the receiver $R\left(X_{s,k+1},X_{r,k+1}\right)$; (2) for the spoofer, it includes the virtual distance $R\left(X_{k+1}^{i},X_{s,k+1}\right)$, which is controlled by the generator’s hardware and software and the physical distance between the spoofer and the receiver $R\left(X_{s,k+1},X_{r,k+1}\right)$. We have $R\left(X_{k+1}^{i},X_{p,k+1}\right)=R\left(X_{k+1}^{i},X_{s,k+1}\right)$. For the algorithm proposed in this paper, the term $R\left(X_{s,k+1},X_{r,k+1}\right)$ is removed due to the difference, while for the traditional least squares method, the term $R\left(X_{s,k+1},X_{r,k+1}\right)$ can be included inside the receiver clock offset term $\delta t_{r,k+1}\times c$.

By comparing Equations (13) and (15), we can see that the only difference is the clock offset term $\delta t_{r,k+1}\times c$. In addition, the method proposed in this paper needs two epochs ($t_{k}$ and $t_{k+1}$). Both of the clock offset term and the result at $t_{k}$ will affect our method’s result at $t_{k+1}$. According to the above analysis, we can write the method as: the position obtained at $t_{k+1}$ is equal to the position at $t_{k}$ plus the position deviation caused by the change in pseudorange between $t_{k}$ and $t_{k+1}$, plus the position deviation caused by the clock offset term. For the position result at $t_{k}$, when the position deviation of the two algorithms at $t_{k}$ is less than the threshold, the traditional least squares method’s result at $t_{k}$ is used to calculate the position result at $t_{k+1}$. When the position deviation of the two algorithms at $t_{k}$ is greater than the threshold, our method’s result at $t_{k}$ is used to calculate the position result at $t_{k+1}$.

To sum up,

- When the traditional least squares method’s result at $t_{k}$ is used, the position at $t_{k+1}$ can be written as: the position obtained at $t_{k+1}$ is equal to the position at $t_{k}$, plus the position deviation caused by the change in the pseudorange between $t_{k}$ and $t_{k+1}$, plus the position deviation caused by $\delta t_{r,k+1}\times c$. From the simulation result in Section 3, we can see that the maximum equivalent distance of the clock offset is $10^{-6}\times c$, which is small enough to ignore its influence, and the position results of the two algorithms are approximately equal.
  (16) $$\left\{\begin{array}{c} x_{k+1,LS}\approx x_{k+1,SDA}\\ y_{k+1,LS}\approx y_{k+1,SDA}\\ z_{k+1,LS}\approx z_{k+1,SDA} \end{array}\right.$$
- When our method’s result at $t_{k},t_{k-1},\ldots t_{k-N+1}$ is used and the traditional least squares method’s result at $t_{k-N}$ is used, the position at $t_{k+1}$ can be written as: the position obtained at $t_{k+1}$ is equal to the position at $t_{k}$, plus the position deviation caused by the change in pseudorange between $t_{k}$ and $t_{k+1}$, plus the position deviation caused by $\sum_{T=k-N+1}^{T=k+1}\delta t_{r,T}\times c$. From the above analysis, we can see that, even though the influence of the clock offset at one epoch is small, the position deviation caused by the clock offset accumulates along with the existence of the spoofing signal. When the position deviation caused by $\sum_{T=k-N+1}^{T=k+1}\delta t_{r,T}\times c$ is greater than the threshold, the inequality of the two algorithms can be obtained.
  (17) $$\left\{\begin{array}{c} x_{k+1,LS}\neq x_{k+1,SDA}\\ y_{k+1,LS}\neq y_{k+1,SDA}\\ z_{k+1,LS}\neq z_{k+1,SDA} \end{array}\right.$$

### 2.2. Setting Parameters of the Proposed Algorithm

If the spoofing signal is present at the beginning of the data collection, the calculation generally starts from the second epoch. Therefore, the position value at the first epoch needs to be set. When the receiver is in a dynamic state, the traditional least squares method’s result at the first epoch is adopted and set as the initial value. When the receiver is in a static state, we always need to set the initial position value as the vehicle’s real position to guarantee that the algorithm is valid.

In addition, we need at least four satellites for the vehicle positioning in the actual calculation of each epoch. We need to select the satellite before the calculation to ensure the accuracy of the navigation and positioning algorithm. Different schemes can be adopted, such as the comprehensive geometric dilution of precision (GDOP) minimum. In this paper, we use the satellite with C/No greater than or equal to 45 dB-Hz in the calculation. If the number of satellites is smaller than four when this constraint is applied (usually in dynamic scenarios), it is appropriate to reduce the value of C/No. For example, for the dynamic position spoofing scenario in the Texas spoofing test battery, we select the satellite with C/No greater than or equal to 40 dB-Hz in the calculation.

The existence of measurement noises, correction residuals, differential calculus and the receiver clock offset’s influence makes the traditional least squares method’s result inconsistent with the spoofing detection algorithm’s result when the signal is real. Therefore, it is necessary to set a reference threshold value. When the position deviation is less than the threshold value, the signal is real, otherwise it is spoofing. As this is an empirical method, the threshold value should be fixed in advance. We determine the threshold value mainly by some prior experiments. In these experiments, the acceptable positioning error, the acceptable false alarm rate and the missed detection rate are considered. When the signal is real, a lower threshold will increase the false alarm rate; while when the signal is spoofing, a higher threshold will increase the missed detection rate. For example, for the static position spoofing scenario in Section 3, when the threshold value is set as 3.5 m, the false alarm rate is 4.7% and the missed detection rate is 1.16%; when the threshold value is set as 5 m, the false alarm rate is 0% and the missed detection rate is 1.55%; when the threshold value is set as 7 m, the false alarm rate is 0% and the missed detection rate is 2.17%. To balance the positioning error, the false alarm rate and the missed detection rate, we chose 5 m and 10 m respectively for the static scenarios and the dynamic scenarios.

### 2.3. Algorithm Summary and Flowchart

**Figure 2:**
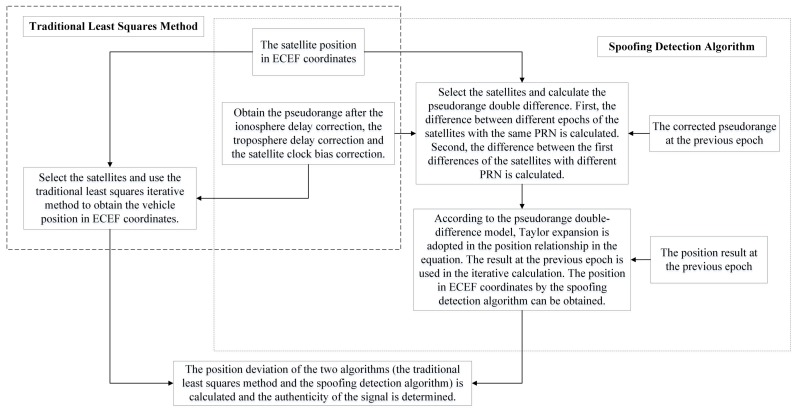
The detailed spoofing detection process.

**Figure 3:**
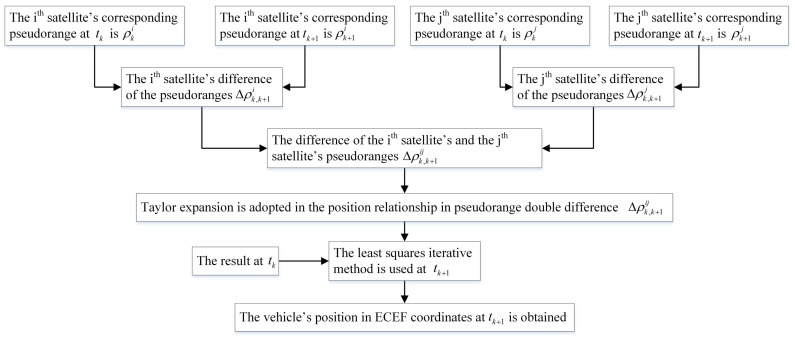
Pseudorange double-difference model establishment and solution.

To sum up, the proposed algorithm has three steps: (1) pseudorange differences calculation; (2) iterative solution; (3) comparison with the traditional least squares method. The conclusion can be drawn as: the spoofing detection algorithm’s result approximates the traditional least squares method’s result when the signal is real. Otherwise, the results of these two algorithms are different. Based on this, the authenticity of the signal can be determined. To implement the algorithm, we need to get the pseudorange and the ephemeris from the GNSS receiver. The satellite position is determined based on the information of the ephemeris. Using the information of the pseudorange, the difference calculation and the iterative solution are carried out. Explicit algorithm flows are given in Figure 2 and Figure 3. In Figure 2, the detailed spoofing detection process is presented. In Figure 3, we give the analysis explanation of the pseudorange double-difference model establishment and solution.

When there are *n* satellites with the same pseudo-random noise (PRN) number at $t_{k}$ and $t_{k+1}$, the first difference calculation needs to be executed once, and the second difference calculation needs to be executed $\left(n-1\right)$ times. Therefore, the algorithm complexity is $O\left(n\right)$.

## 3. Simulation Tests

To verify the feasibility and effectiveness of the algorithm, different kinds of test datasets, including the university test dataset, the Beidou Open Laboratory test dataset and the Texas spoofing test battery (TEXBAT), are used in the simulation. We generated the first two datasets at our university and Beidou Open Laboratory, respectively, and the TEXBAT comprised the only public spoofing test datasets published by The University of Texas at Austin [26,27,28,29]. The data generation of these datasets is described in the following subsections. The performance of the algorithm is also verified in the dynamic whole-time duration position spoofing scenario. In all spoofing scenarios, all satellites are spoofed, and there is only one spoofer. Other characteristics of different scenarios are summarized in Table 1. The false alarm rate and the missed detection rate in each scenario are calculated. The false alarm rate indicates the probability that the algorithm misjudges the real signal as the spoofing one. The missed detection rate indicates the probability that the algorithm misjudges the spoofing signal as the real one.

### 3.1. University Test

**Figure 4:**
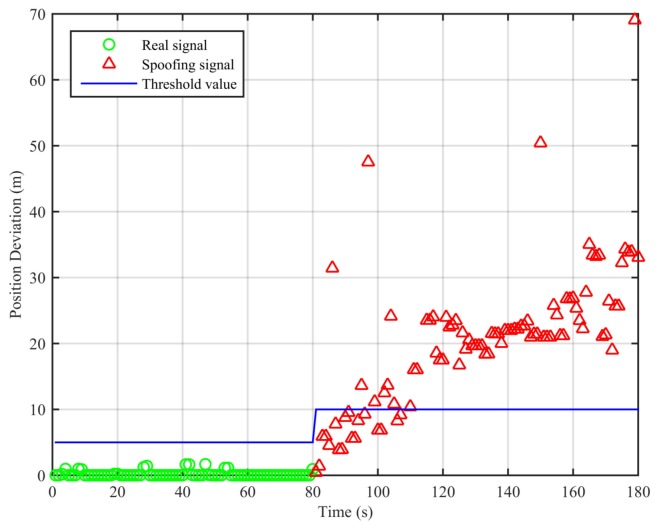
Position deviations of the two algorithms (Experiment No. 1).

In this scenario, the receiver is in a static state first, and it receives the real signal. This process lasts about 80 s. Then, the signal transponder is turned on. The receiver is held by hand and approaches the signal transponder’s transmitting antenna slowly. This process is repeated twice and lasts about 100 s. We adopt the algorithm proposed in this paper and the traditional least squares method to analyze the collected data; the result is shown in Figure 4.

From Figure 4, we can see that the position deviations are smaller than the threshold value when the signal is real. When the signal transponder is turned on, the receiver starts to receive the replayed signal, and the position deviations start to increase and become larger than the threshold value. Based on these differences, we can determine the existence of the spoofing signal. The false alarm rate when the signal is real is 0%, and the missed detection rate when the signal is spoofing is 18%. The missed detections mainly occur within the short period after the signal is switched from real to spoofing. In this period, the position deviations caused by the spoofing signal are smaller than the threshold value.

### 3.2. Beidou Open Laboratory Test

To further verify the effectiveness of the algorithm, we conducted Experiment No. 2 in Beidou Open Laboratory. We used the antenna on the roof of the building to introduce the satellite’s signal into the room and assumed this signal to be real. The signal is input into the spoofing signal simulator, and the spoofing signal is output after the computer calculation. The output spoofing signal from the simulator moves circularly. The receiver is adopted in the whole duration. In this duration, the signal is real at first, and this process lasts about 90 s. Then, the spoofing signal simulator is turned on. The absolute power advantage guarantees that the receiver can receive the spoofing signal. The circular motion signal is then acquired. This process lasts about 135 s. Finally, the simulator is turned off, and the signal becomes real. This process lasts about 90 s.

**Figure 5:**
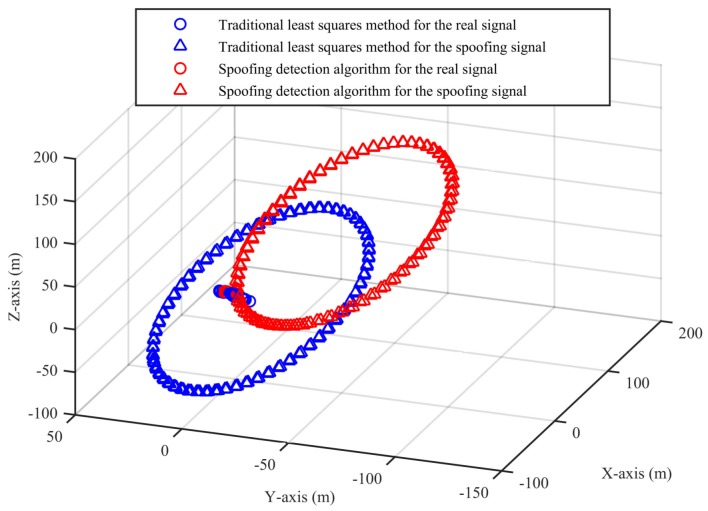
Three-dimensional results of the two algorithms (Experiment No. 2).

We apply the traditional least squares method and the spoofing detection algorithm to the datasets and use (−2,185,955.407, 5,181,417.961, 2,999,272.014) as a reference point for coordinate transformation in the results shown in Figure 5.

**Figure 6:**
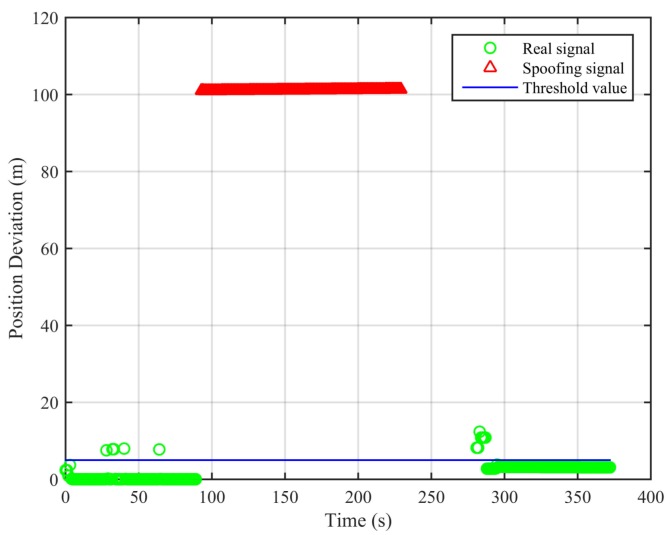
Position deviations of the two algorithms (Experiment No. 2).

From Figure 5, we can see that, for the real signal, the results of the two algorithms are basically the same. For the spoofing signal with circular motion, the difference of the results of the two algorithms is obvious. In Figure 6, we can see that the position deviations in the first segment and the third segment are always small. The position deviations perform a significant change and remain large when the signal is switched from real to spoofing. These differences can help us to validate the signal authenticity. The false alarm rate when the signal is real is 2.91%, and the missed detection rate when the signal is spoofing is 2.04%.

### 3.3. The Texas Spoofing Test Battery

Finally, we adopt the only public spoofing test datasets, the Texas spoofing test battery (TEXBAT), to verify the detection performance of the algorithm. This involves eight separate spoofing scenarios and two clean scenarios. In this paper, we mainly focus on the positioning terminal. Therefore, for the spoofing experiments, we adopt the static position spoofing scenario and the dynamic position spoofing scenario. As a reference, we also adopt the two clean scenarios, which are the clean static scenario and the clean dynamic scenario. The remaining five datasets are the static or dynamic time spoofing scenarios and the signal switch scenario. Time spoofing scenarios focus mainly on the timing terminal.

In the clean static scenario, the receiver is placed on the roof of the Aerospace Engineering Building at the University of Texas to receive and record the real signal. In the clean dynamic scenario, the receiver platform is dynamic rather than static. The clean dynamic dataset was originally recorded by an antenna on a traveling vehicle. The static position spoofing scenario is based on the clean static scenario. The static signal is real in the first segment (around 170 s). Then, the receiver captures the spoofing signal with the power advantage of 0.4 dB higher than the real one. The spoofing signal slowly drives the receiver off the real position, and the ultimate bias is an offset of 600 m in the Z direction. In the dynamic position spoofing scenario, which is based on the clean dynamic scenario, the receiver is in a dynamic state. The signal is real at first. The spoofing signal slowly drives the receiver off the real position with a 0.8-dB power advantage in about 100 s. The ultimate bias is also an offset of 600 m in the Z direction.

For the TEXBAT datasets, we replay them with National Instruments (NI) equipment (NI equipment model numbers: NI PXIe-5450: 400 MS/s In-phase/Quadrature (I/Q) Signal Generator, NI PXIe-5611: I/Q Vector Modulator, NI PXI-5652: Radio Frequency (RF) Signal Generator) at Shanghai Advanced Research Institute, Chinese Academy of Sciences. The traditional least squares method and the spoofing detection algorithm proposed in this paper are used in the experiments.

**Figure 7:**
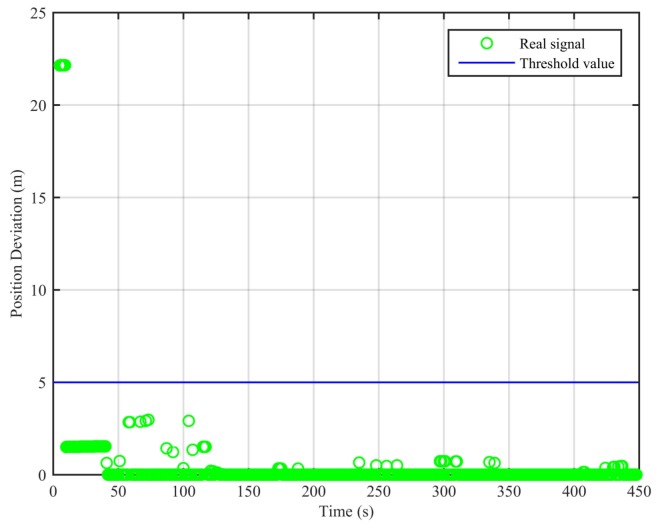
Position deviations of the two algorithms (clean static scenario).

**Figure 8:**
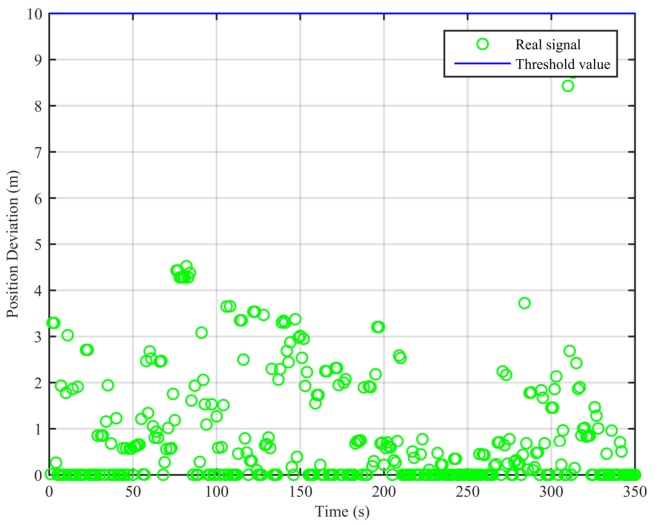
Position deviations of the two algorithms (clean dynamic scenario).

**Figure 9:**
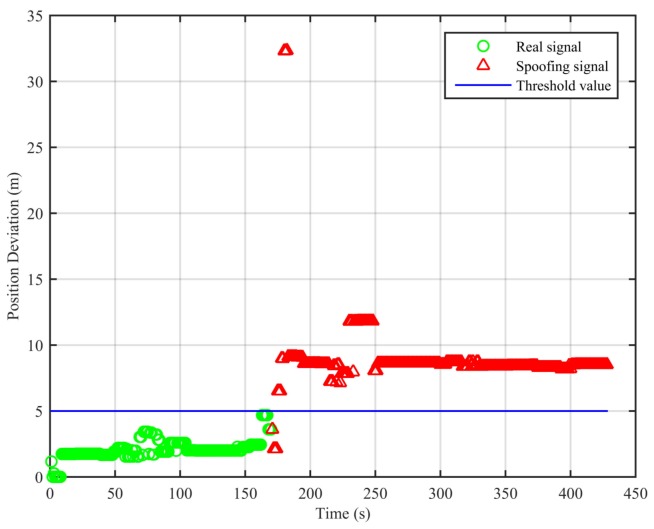
Position deviations of the two algorithms (static position spoofing scenario).

**Figure 10:**
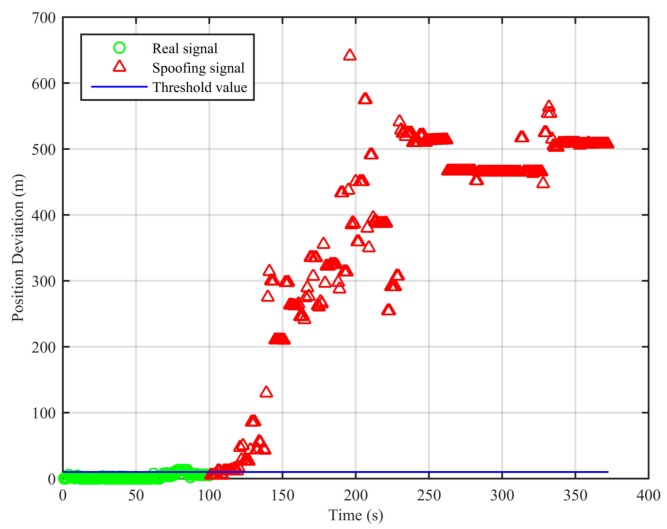
Position deviations of the two algorithms (dynamic position spoofing scenario).

Figure 7, Figure 8, Figure 9 and Figure 10 show the position deviations of the two algorithms in the clean static scenario, the clean dynamic scenario, the static position spoofing scenario and the dynamic position spoofing scenario, respectively.

As can be seen from Figure 7, the real signal is received in the clean static scenario, and the position deviations are always small. The false alarm rate when the signal is real is 1.12%. The false alarms mainly occur at the beginning, due to the code smoothing process embedded in the receiver. In Figure 8, the real signal is received in the clean dynamic scenario. Though the position deviations in Figure 8 are larger than those in Figure 7, no big jump occurs in the whole duration. The false alarm rate when the signal is real is 0%. In Figure 9 and Figure 10, we can also observe that there exist obvious jumps in the position deviations of the two algorithms when the signal is switched from real to spoofing and that these jumps remain in the following duration. In the static position spoofing scenario, the false alarm rate when the signal is real is 0%, and the missed detection rate when the signal is spoofing is 1.55%. In the dynamic position spoofing scenario, the false alarm rate when the signal is real is 8%, and the missed detection rate when the signal is spoofing is 2.57%.

### 3.4. Dynamic Whole-Time Duration Spoofing Scenario

**Figure 11:**
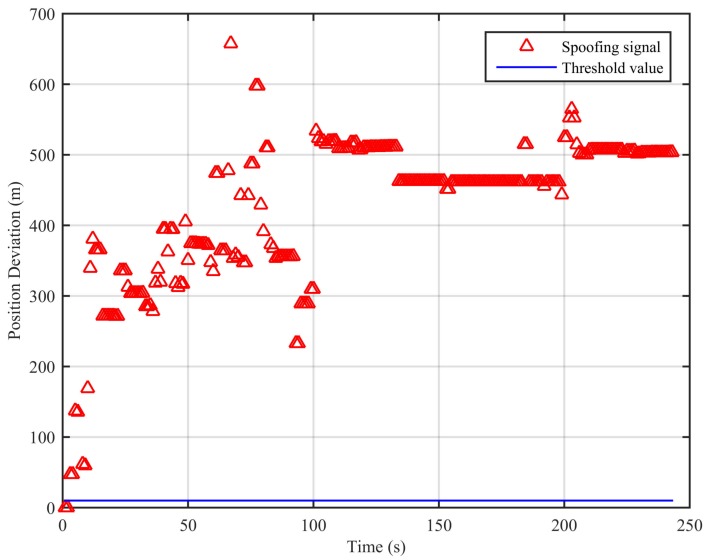
Position deviations of the two algorithms (dynamic whole-time duration position spoofing scenario).

Further, we select the spoofing part of the dynamic position spoofing scenario in the TEXBAT datasets to construct the dynamic whole-time duration spoofing scenario to verify the performance of the algorithm. The simulation results are shown in Figure 11.

The traditional least squares method’s result at the first epoch is used as the initial value in the calculation. From Figure 11, we can see that the position deviations are larger than the threshold, and this indicates the existence of the spoofing signal. In this scenario, the missed detection rate when the signal is spoofing is 0.82%. This proves that the proposed spoofing detection algorithm is effective for the dynamic whole-time duration position spoofing scenario.

For the spoofing scenarios, the positions obtained by the traditional least squares method and the spoofing detection algorithm are different, and the position deviations will always be larger than the threshold value as long as the spoofing signal exists. The authenticity of the signal can then be determined. The spoofing detection algorithm’s results do not represent the receiver’s real position, thus the position deviations do not represent the position bias caused by the spoofing signal. Therefore, we cannot see the effect of the 600-m offset. If it is only solved by the traditional least squares method, we can observe that the receiver is slowly driven by the spoofing signal and reaches its final offset of 600 m.

### 3.5. Comparison with Other Methods

**Figure 12:**
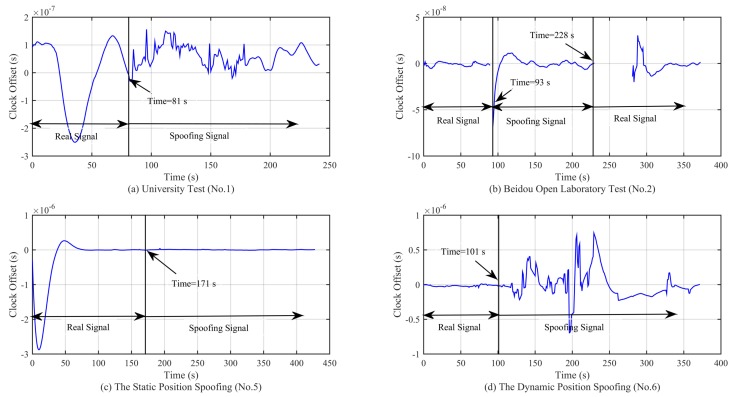
Curve of the clock offset in different experimental scenarios.

**Figure 13:**
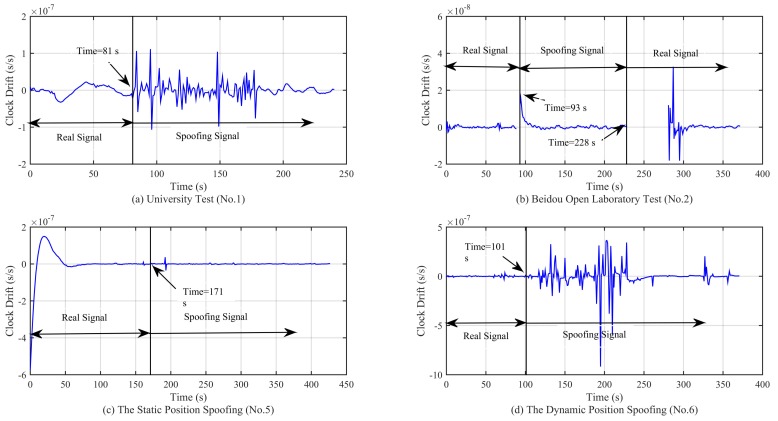
Curve of the clock drift in different experimental scenarios.

**Figure 14:**
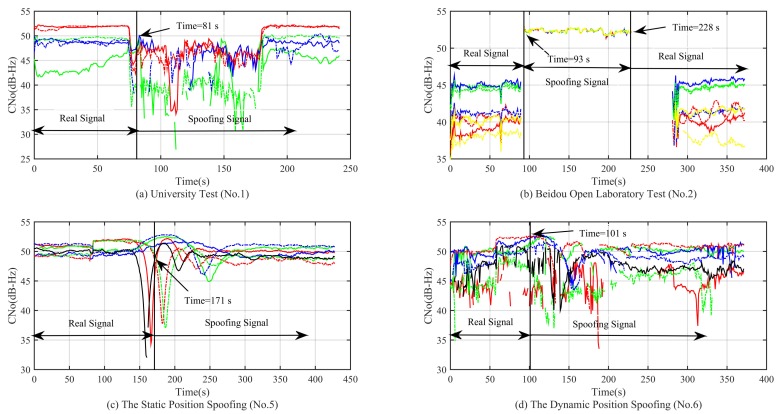
Curve of the carrier noise power ratio (C/No) in different experimental scenarios.

**Figure 15:**
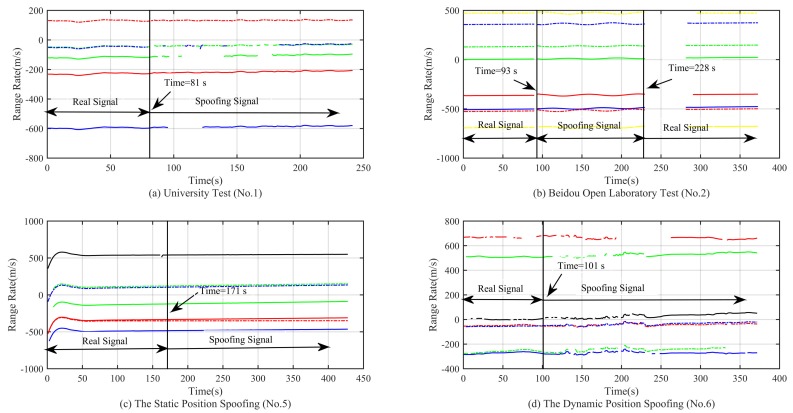
Curve of the range rates in different experimental scenarios.

To further evaluate the performance of the algorithm, we select the related methods (range rates jump detection, C/No jump detection, the clock offset and the clock drift jump detection) from [1] in the simulation, and the results are shown in Figure 12, Figure 13, Figure 14 and Figure 15.

As shown on Figure 12a,c, there is a slow convergence at the beginning; this is due to the code smoothing process within the receiver. From the simulation results, we can see that when the spoofing signal appears, the clock offset presents an oscillating state, and the amplitude of the oscillation is related to the motion state. For example, Figure 12a,d shows the cases where the receiver is in a moving state and the clock offset oscillation range is larger than that of Figure 12b,c. In the Beidou Open Laboratory test, the large oscillation only occurs in the process of signal switching. When the signal is locked in the spoofing signal, the clock offset oscillation is not so obvious, and the spoofing signal has completely taken over the receiver at this time. In addition, it can be seen from the four subfigures in Figure 12 that the clock offset is not the same magnitude, which limits the application of the method based on the jump detection of the clock offset, as the detection threshold needs to be set in advance. This method cannot be applied to the scenario in which the signal is spoofing from the beginning. From the simulation results in Figure 13, we can see that the clock drift shows similar characteristics as the clock offset. The range and magnitude of the oscillation will limit the application of this method. Moreover, as can be seen from Figure 12c and Figure 13c, there is no obvious change in the clock offset and the clock drift, which poses a challenge for the signal detection based on the method in [1].

From the simulation result in Figure 14a,b, we can see that the C/No has a relatively significant change when the spoofing signal is acquired. At this time, we can use the method of C/No jump detection. For the static and dynamic position spoofing scenario, the spoofing signal is switched by a 0.4-dB and a 0.8-dB power advantage, respectively. Therefore, we cannot observe any obvious changes. The method of C/No jump detection is invalid for these two kinds of spoofing scenarios. From the simulation result in Figure 15, we can see that none of the changes of range rates for these four kinds of spoofing scenarios is obvious. Therefore, the method of range rate jump detection is invalid.

We summarize the detection ability of different methods in Table 2. From Table 2, we can see that range rates jump detection is invalid for all scenarios in this paper (in fact, it is only effective for the simplest spoofing attacks). The other three methods (C/No jump detection, clock offset jump detection, clock drift jump detection) would be effective at detecting only some of the spoofing attacks. Our algorithm, proposed in this paper, shows effective detection performance for all the above scenarios.

## 4. Conclusions

In this paper, we propose an effective spoofing detection algorithm. We establish the pseudorange double-difference model. The ionosphere delay correction, the troposphere delay correction and the satellite clock offset correction are considered and used to correct the pseudorange. Taylor expansion is applied to the position relationship between the vehicle and the satellite. To guarantee the performance of the algorithm, we give the parameter setting of the proposed algorithm. Different kinds of test datasets are used to verify the effectiveness and the feasibility of the algorithm. From the simulation results, we can verify the advantageous performance of the algorithm.

The algorithm has the advantage of simplicity for use in engineering applications. First, the requirement for the equipment is low compared to multi-antenna detection algorithms. Only one single receiver is required, and the hardware of the receiver does not need to be changed. Second, the requirement for the measurement information is low compared to multi-algorithm fusion detection methods. Only the pseudorange is required. Last, the algorithm has a low complexity. The calculation of the pseudoranges is executed only twice before the iterative calculation.

## Figures and Tables

**Table 1:** The characteristics of different experiments.

Experiment	Scenario Type	Receiver State	Signal Type
No. 1 (University Test)	meaconing attack	static and dynamic	real and spoofing
No. 2 (Beidou Open Laboratory Test)	simplistic attack	static	real and spoofing
No. 3 (The Texas Spoofing Test Battery)	real	static	real
No. 4 (The Texas Spoofing Test Battery)	real	dynamic	real
No. 5 (The Texas Spoofing Test Battery)	intermediate attack	static	real and spoofing
No. 6 (The Texas Spoofing Test Battery)	intermediate attack	dynamic	real and spoofing
No. 7 (The Texas Spoofing Test Battery)	intermediate attack	dynamic	spoofing

**Table 2:** Different methods’ detection ability.

	No. 1	No. 2	No. 5	No. 6
Range Rates Jump Detection				
C/No Jump Detection	Detects	Detects		
Clock Offset Jump Detection	Detects			Detects
Clock Drift Jump Detection	Detects			Detects
Algorithm in This Paper	Detects	Detects	Detects	Detects

## Abbreviations

The following abbreviations are used in this paper:

GNSS	Global navigation satellite system
TEXBAT	Texas spoofing test battery
PRN	Pseudo-random noise
ECEF	Earth-centered Earth-fixed
GDOP	Geometric dilution of precision
NI	National Instruments
I/Q	In-phase/quadrature
RF	Radio frequency
P code	Precision code
C/A code	Coarse/acquisition code
C/No	Carrier noise power ratio

## References

[1] Wen H.Q., Huang P.Y.-R., Dyer J., Archinal A., Fagan J. Countermeasures for GPS Signal Spoofing. Proceedings of the 18th International Technical Meeting of the Satellite Division of the Institute of Navigation (ION GNSS 2005).

[2] Humphreys T.E., Ledvina B.M., Psiaki M.L., O’Hanlon B.W., Kintner P.M. Assessing the Spoofing Threat: Development of a Portable GPS Civilian Spoofer. Proceedings of the 21st International Technical Meeting of the Satellite Division of the Institute of Navigation (ION GNSS 2008).

[3] U.S. Military Definition of Meaconing. http://www.dtic.mil/doctrine/jel/doddict/data/m/03301.html.

[4] Huang L., Lv Z.C., Wang F.X. (2012). Spoofing pattern research on GNSS receivers. J. Astronaut..

[5] Dehghanian V., Nielsen J., Lachapelle G. (2012). GNSS spoofing detection based on signal power measurements: Statistical analysis. Int. J. Navig. Obs..

[6] Jafarnia-Jahromi A., Broumandan A., Nielsen J., Lachapelle G. (2014). Pre-despreading authenticity verification for GPS L1 C/A signals. Navig. J. Inst. Navig..

[7] Borio D. (2013). PANOVA tests and their application to GNSS spoofing detection. IEEE Trans. Aerosp. Electron. Syst..

[8] Zhang Y.T., Wang L., Wang W.Y., Lu D., Wu R.B. Spoofing jamming suppression techniques for GPS based on DoA estimating. Proceedings of the China Satellite Navigation Conference (CSNC 2014).

[9] Broumandan A., Jafarnia-Jahromi A., Daneshmand S., Lachapelle G. Effect of Tracking Parameters on GNSS Receiver Vulnerability to Spoofing Attack. Proceedings of the 29th International Technical Meeting of the Satellite Division of the Institute of Navigation (ION GNSS+ 2016).

[10] Wang W.Y., Chen G., Wu R.B., Lu D., Wang L. A low-complexity spoofing detection and suppression approach for ADS-B. Proceedings of the Integrated Communications Navigation and Surveillance (ICNS) Conference.

[11] Borio D., Gioia C. (2016). A sum-of-squares approach to GNSS spoofing detection. IEEE Trans. Aerosp. Electron. Syst..

[12] Radin D.S., Swaszek P.F., Seals K.C., Hartnet R.J. GNSS Spoof Detection Based upon Pseudoranges from Multiple Receivers. Proceedings of the 2015 International Technical Meeting of the Institute of Navigation.

[13] Broumandan A., Jafarnia-Jahromi A., Lachapelle G. (2015). Spoofing detection, classification and cancelation (SDCC) receiver architecture for a moving GNSS receiver. GPS Solut..

[14] Psiaki M.L., O’ Hanlon B.W., Bhatti J.A., Shepard D.P., Humphreys T.E. (2013). GPS spoofing detection via dual-receiver correlation of military signals. IEEE Trans. Aerosp. Electron. Syst..

[15] Lee J.H., Kwon K.C., An D.S., Shim D.S. (2015). GPS Spoofing Detection Using Accelerometers and Performance Analysis with Probability of Detection. Int. J. Control Autom. Syst..

[16] White N.A., Maybeck P.S., DeVilbiss S.L. (1998). Detection of Interference/Jamming and Spoofing in a DGPS-Aided Inertial System. IEEE Trans. Aerosp. Electron. Syst..

[17] Gamba M.T., Truong M.D., Motella B., Falletti E., Ta T.H. (2017). Hypothesis testing methods to detect spoofing attacks: A test against the TEXBAT datasets. GPS Solut..

[18] Wang F., Li H., Lu M.Q. (2017). GNSS Spoofing Detection and Mitigation Based on Maximum Likelihood Estimation. Sensors.

[19] Manfredini E.G., Dovis F. (2016). On the Use of a Feedback Tracking Architecture for Satellite Navigation Spoofing Detection. Sensors.

[20] Kim T.H., Sin C.S., Lee S., Kim J.H. Analysis of effect of anti-spoofing signal for mitigating to spoofing in GPS L1 signal. Proceedings of the 2013 13th International Conference on Control, Automation and Systems (ICCAS).

[21] Pozzobon O., Canzian L., Danieletto M., Chiara A.D. Anti-spoofing and open GNSS signal authentication with signal authentication sequences. Proceedings of the IEEE Satellite Navigation Technologies and European Workshop on Gnss Signals and Signal, Noordwijk, The Netherlands.

[22] Li W.T., Huang Z.G., Lang R.L., Qin H.L., Zhou K., Cao Y.B. (2016). A Real-Time Interference Monitoring Technique for GNSS Based on a Twin Support Vector Machine Method. Sensors.

[23] Tao H.Q., Li H., Lu M.Q. (2016). A Method of Detections’ Fusion for GNSS Anti-Spoofing. Sensors.

[24] Zhang Z.J., Zhan X.Q. (2016). GNSS Spoofing Network Monitoring Based on Differential Pseudorange. Sensors.

[25] Groves P.D. (2013). Navigation Processor. Principles of GNSS, Inertial, and Multisensor Integrated Navigation Systems.

[26] Humphreys T.E., Bhatti J.A., Shepard D.P., Wesson K.D. The Texas Spoofing Test Battery: Toward a Standard for Evaluating GPS Signal Authentication Techniques. Proceedings of the 25th International Technical Meeting of the Satellite Division of the Institute of Navigation (ION GNSS+ 2012).

[27] Humphreys T.E. Texbat Data Sets 7 and 8. Technical Report. http://radionavlab.ae.utexas.edu/datastore/texbat/texbat_ds7_and_ds8.pdf..

[28] Lemmenes A., Corbell P., Gunawardena S. Detailed Analysis of the TEXBAT Datasets Using a High Fidelity Software GPS Receiver. Proceedings of the 29th International Technical Meeting of the Satellite Division of the Institute of Navigation (ION GNSS+ 2016).

[29] The TEXBAT Datasets. http://radionavlab.ae.utexas.edu/datastore/texbat/.

